# Characteristics, clinical course, and outcomes of homeless and non-homeless patients admitted to ICU: A retrospective cohort study

**DOI:** 10.1371/journal.pone.0179207

**Published:** 2017-06-12

**Authors:** Orla M. Smith, Clarence Chant, Karen E. A. Burns, Maninder Kaur, Said Ashraf, Claudia C. DosSantos, Stephen W. Hwang, Jan O. Friedrich

**Affiliations:** 1 Critical Care Department, St. Michael’s Hospital, Toronto, Canada; 2 Li Ka Shing Knowledge Institute, St. Michael’s Hospital, Toronto, Canada; 3 Lawrence S. Bloomberg Faculty of Nursing, University of Toronto, Toronto, Canada; 4 Pharmacy Department, St. Michael’s Hospital, Toronto, Canada; 5 Department of Medicine, St. Michael’s Hospital and University of Toronto, Toronto, Canada; 6 Interdepartmental Division of Critical Care Medicine, University of Toronto, Toronto, Canada; 7 Centre for Urban Health Solutions, St. Michael’s Hospital, Toronto, Canada; Universidade do Extremo Sul Catarinense, BRAZIL

## Abstract

**Background:**

Little is known about homeless patients in intensive care units (ICUs).

**Objectives:**

To compare clinical characteristics, treatments, and outcomes of homeless to non-homeless patients admitted to four ICUs in a large inner-city academic hospital.

**Methods:**

63 randomly-selected homeless compared to 63 age-, sex-, and admitting-ICU-matched non-homeless patients.

**Results:**

Compared to matched non-homeless, homeless patients (average age 48±12 years, 90% male, 87% admitted by ambulance, 56% mechanically ventilated, average APACHE II 17) had similar comorbidities and illness severity except for increased alcohol (70% vs 17%,p<0.001) and illicit drug(46% vs 8%,p<0.001) use and less documented hypertension (16% vs 40%,p = 0.005) or prescription medications (48% vs 67%,p<0.05). Intensity of ICU interventions was similar except for higher thiamine (71% vs 21%,p<0.0001) and nicotine (38% vs 14%,p = 0.004) prescriptions. Homeless patients exhibited significantly lower Glasgow Coma Scores and significantly more bacterial respiratory cultures. Longer durations of antibiotics, vasopressors/inotropes, ventilation, ICU and hospital lengths of stay were not statistically different, but homeless patients had higher hospital mortality (29% vs 8%,p = 0.005). Review of all deaths disclosed that withdrawal of life-sustaining therapy occurred in similar clinical circumstances and proportions in both groups, regardless of family involvement. Using multivariable logistic regression, homelessness did not appear to be an independent predictor of hospital mortality.

**Conclusions:**

Homeless patients, admitted to ICU matched to non-homeless patients by age and sex (characteristics most commonly used by clinicians), have higher hospital mortality despite similar comorbidities and illness severity. Trends to longer durations of life supports may have contributed to the higher mortality. Additional research is required to validate this higher mortality and develop strategies to improve outcomes in this vulnerable population.

## Introduction

Homelessness is a serious social and public health problem. Prevalence estimates indicate that at least 150,000 people were homeless in Canada in 2009 [1] and 1.5 million people were homeless in the United States in 2012 [2]. A comprehensive survey conducted by the City of Toronto, Canada in 2013 estimated a point prevalence of 5,253 homeless people in Toronto on one night, corresponding to 18.8 homeless people per 10,000 population [3]. Homeless persons have disproportionately higher rates of infectious diseases, chronic diseases, mental illness, substance use, and intentional and unintentional injuries [4,5,6]. Moreover, homelessness is associated with earlier onset of health problems that are otherwise more commonly seen in geriatric populations including hypertension [7].

Homeless individuals are admitted to hospital more often than the general population [8,9]. This may be due to suboptimal access to preventive health care and medical treatment, higher levels of comorbid conditions, and presentation for acute care when symptoms are more severe. In addition, homeless people stay in hospital longer than housed individuals, as having no residence can deter timely hospital discharge [8,9]. Homeless people admitted to hospital tend to be younger than non-homeless comparators and are more frequently admitted for medical or psychiatric conditions versus surgical conditions [10]. Costs associated with the care of homeless individuals in hospital are significantly greater than those incurred by age- and sex-matched individuals [10]. However, the course of illness and use of health care resources for people who are homeless admitted to the intensive care unit (ICU) are largely unknown. A recent systematic review noted the paucity of research related to the course and outcomes of homeless patients in the ICU [11]. In a subsequently published propensity-matched cohort from France, homelessness was not associated with ICU or hospital mortality but was associated with significantly longer ICU and hospital stays [12]. Given this higher use of hospital and intensive care resources and that intensive care is a limited and costly resource, a better understanding of the needs and outcomes of homeless critically ill patients would be useful for health system planning and improvement. We aimed to describe the clinical characteristics, course of critical illness, decision-making, and ICU and hospital outcomes, of homeless persons admitted to the ICUs in a large urban academic hospital in Canada, and to compare these characteristics and outcomes to a matched group of non-homeless critically ill patients.

## Methods

We conducted a retrospective chart review of homeless persons who were admitted to any of four ICUs [Medical-Surgical Intensive Care Unit (MSICU), Trauma-Neurosurgical Intensive Care Unit (TNICU), Cardiovascular Intensive Care Unit (CVICU), and Cardiac Intensive Care Unit (CICU)] at St. Michael’s Hospital, Toronto, Canada, from January 1, 2009 to December 31, 2011. The study hospital is located in the inner city of Toronto, a city of around 5 million people, and has 462 acute care beds with 68 ICU beds. A teaching hospital affiliated with the University of Toronto, the hospital serves as a regional referral centre for a number of specialty services, and our Inner City Health program cares for many of Toronto’s poor and homeless. Homeless patients were identified using an indicator variable in the hospital’s administrative database that was triggered when patients were registered as having no fixed address, a residential address corresponding to a local homeless shelter, or a dummy postal code reserved for homeless patients [10]. Using a random number generator, we randomly selected patients from identified cases, choosing a sample of 100 patients based on feasibility of data collection. Each homeless patient was matched to a non-homeless patient based on pre-defined criteria: calendar year of admission (2009, 2010, 2011), sex, age (exact year), and ICU to which the patient was admitted (i.e., MSICU, TNICU, CVICU, or CCU). If multiple matches within the same calendar year were possible, we selected a matching patient by using the closest admission date. If no match was found, age range could be broadened progressively from ±1 year to ±5 years, with matching to any of the 3 possible admission years. We chose age and sex as our primary matching criteria since these are most commonly used by clinicians on initial assessment, with age in particular likely to be the most important predictor of outcome. We added year of admission to minimize effects of temporal trends in outcomes over the course of the study, and type of ICU because diagnoses and outcomes differ substantially between the ICUs in our hospital.

Hospital records of identified patients were reviewed by one of two authors (MK, SA) to confirm the patient had been admitted into an ICU bed at St. Michael’s Hospital. Charts of all patients identified as homeless were also examined by a third reviewer (SH) with experience in research on homelessness to ascertain if: 1) housing status was identified correctly, and 2) the patient resided in a shelter or on the street prior to admission. Homelessness was defined as being unsheltered or emergency sheltered, consistent with the categorization scheme used in the Canadian definition of homelessness [13]. The same third reviewer also reviewed charts of all the matched non-homeless patients to ensure that they were indeed housed. We developed a standardized case report form to capture data at ICU admission, during the course of the ICU stay (Days 1, 3, and 7), and at ICU and hospital discharge. If patients were admitted to the ICU more than once during their hospitalization, we recorded data for the first ICU admission only.

Data abstraction was performed by two authors (MK, SA) using a standardized data dictionary. Quality assurance in data abstraction was verified by two other authors (OMS, CC) for the first 10 charts prior to proceeding with the remaining data abstraction. A random sample of 5% of charts was also audited by one additional author to ensure accuracy. For all patient deaths, circumstances surrounding death were independently reviewed by two authors (OMS, JOF).

Categorical variables are presented as number and percentage for homeless and non-homeless critically ill patients. We present group-specific mean and standard deviations for normally distributed variables, and median and interquartile range or range for non-normally distributed variables. We compared homeless and non-homeless patients using Fisher’s exact test for categorical variables; and unpaired t-test or Wilcoxon-Mann-Whitney test for continuous variables that were normal or skewed, respectively. In a sensitivity analysis, we also conducted paired analyses where appropriate for each matched homeless/non-homeless pair using McNemar’s test (Bowker’s test for contingency tables larger than 2 × 2) for categorical variables, and paired t-tests or Wilcoxon Signed-Rank Test for normal and skewed variables, respectively. As unpaired and paired analyses provided virtually identical p-values for all comparisons, we present the results for unpaired analyses only, which could be performed for all comparisons. We performed statistical calculations using VassarStats (available at www.vassarstats.com), Simple Interactive Statistical Analysis (SISA) for Fisher’s exact test with contingency tables larger than 2 × 4 (available at http://www.quantitativeskills.com/sisa/statistics/fiveby2.htm), and Marginal Homogeneity (MH) Statistical Program (v. 1.2) for Bowker’s test (available at http://john-uebersax.com/stat/mh.htm).

We also conducted multivariable logistic regression analysis using SAS version 9.4 (SAS Institute Inc., Cary, NC, USA) to determine if homelessness was an independent predictor of hospital mortality. All variables with p<0.20 by univariate logistic regression were entered into a multivariable logistic regression model and sequentially removed using backward selection until all remaining variables had p<0.10. All retained variables were then assessed for collinearity or the presence of significant second-order interactions. Discrimination and calibration of models was assessed by the area under the receiver operating characteristic curves and the Hosmer and Lemeshow chi-squared statistic, respectively.

## Results

We identified 369 patients admitted to one of our ICUs from 2009 to 2011 labeled as homeless in our administrative database. A random sample of 100 patients was selected, of which 63 were confirmed homeless. Misidentification of patients as homeless was due to incomplete registration information (i.e. address missing), which could occur if the person had collapsed alone on the street with incomplete identification, or arrival to hospital in critical condition without proper registration. There were a few who were flagged as homeless but on review of the chart, we determined they were living in rooming houses. Patients confirmed to be homeless lived in a shelter (35/63), on the street (2/63), or were homeless with the location where they slept not documented (26/63). Most (90%) homeless patients were male, with an average age of 48 ±12 years and various documented comorbidities (Tables 1 and 2). Just under half (48%) were taking prescription medications, while alcohol and illicit drug use were used by 70% and 46%, respectively, based on the documented history in the medical charts. All admissions were emergent. Most homeless patients (n = 55; 87%) were brought to hospital by emergency medical services (EMS) and admitted to either our MSICU (59%) or TNICU (29%). Their median APACHE II score was 17 (range, 3–37) and 56% of patients were mechanically ventilated.

**Table 1:** Patient characteristics.

Characteristic	Homeless (n = 63)	Non-homeless (n = 63)	p value
Age (years), mean ±SD, range	48 ±12, 22–71	48 ±12, 22–71	1.00*
Male, n (%)	57 (90%)	57 (90%)	1.00*
Weight (kg), mean ±SD, range	77 ±15, 45–120	82 ±17, 54–136	0.08
Comorbidities at ICU admission, n (%)			
Depression	11 (17%)	7 (11%)	0.45
Hypertension	10 (16%)	25 (40%)	0.005
COPD	7 (11%)	3 (5%)	0.32
Diabetes	5 (8%)	5 (8%)	1.00
Arrhythmia	5 (8%)	4 (6%)	1.00
Schizophrenia	5 (8%)	1 (2%)	0.21
Tuberculosis	3 (5%)	0	0.24
Cancer	2 (3%)	6 (10%)	0.27
Gastrointestinal bleeding	2 (3%)	2 (3%)	1.00
Peripheral vascular disease	2 (3%)	2 (3%)	1.00
Congestive heart failure	1 (2%)	2 (3%)	1.00
Severe liver disease	1 (2%)	0	1.00
Pulmonary hypertension	0	1 (2%)	1.00
Total number of comorbidities at ICU admission, median (IQR; range)	1 (0–2; 0–4)	1 (0–2; 0–6)	0.88
Prescription medications at ICU admission	30 (48%)	42 (67%)	0.047
Documented substance use history			
Alcohol	44 (70%)	11 (17%)	<0.001
Illicit drugs	29 (46%)	5 (8%)	<0.001
Smoking	25 (40%)	13 (21%)	0.03
Substitute decision-maker identified at admission	24 (38%)	52 (83%)	<0.001

Notes.

*Variable used for matching.

Table Abbreviations: COPD, chronic obstructive pulmonary disease; ICU, intensive care unit; IQR, interquartile range; kg, kilograms; n, number of patients; SD, standard deviation.

**Table 2:** ICU admission data.

Characteristic	Homeless (n = 63)	Non-homeless (n = 63)	p value
Type of Intensive Care Unit^1^, n (%)			0.98
Medical-Surgical	36 (57%)	35 (56%)	
Trauma-Neurosurgical	19 (30%)	20 (32%)	
Coronary Care	7 (11%)	7 (11%)	
Cardiovascular	1 (2%)	1 (2%)	
Type of Admission^2^, n (%)			0.054
Medical	43 (68%)	42 (67%)	
Trauma	16 (25%)	9 (14%)	
Surgical	4 (6%)	12 (19%)	
Admission Diagnosis^2^, n (%)			0.61
Trauma	16	9	
Neurologic	12	17	
Respiratory	11	9	
Cardiovascular/Vascular	10	10	
Other	6	6	
Metabolic/endocrine	4	2	
Gastrointestinal	2	5	
Sepsis	1	2	
Renal	1	1	
Orthopedic	0	2	
Location prior to admission			0.004
Emergency room	48 (76%)	35 (56%)	
Operating room^3^	10^4^ (13%)	14 (22%)	
Hospital ward	5 (8%)	6^5^ (9%)	
Referring hospital	0	8^6^ (13%)	
Arrival at study hospital via EMS, n (%)	55 (87%)	41 (65%)	0.006
APACHE II, mean ±SD (range)	18 ±8 (3–37)	16 ±9 (1–43)	0.11
MV on ICU admission	35 (56%)	27 (43%)	0.21
Toxicology screen performed, n (%)	47 (75%)	24 (38%)	<0.001
Toxicology screen positive, n (%)	34/47 (72%)	17/24 (71%)	1.00
Toxin^7^, n (%)			
Ethanol	26 (41%)	6 (10%)	<0.001
Benzodiazepine	17 (27%)	10 (16%)	0.19
Opioid	6 (10%)	2 (3%)	0.27
Cannabinoid	6 (10%)	2 (3%)	0.27
Cocaine	5 (8%)	1 (2%)	0.21
Acetaminophen	6 (10%)	0	0.03
Salicylate	5 (8%)	0	0.06

Notes.

^1^ Variable used for matching.

^2^Categorized using APACHE III diagnostic categories.

^3^The post-operative patients are further classified by type of surgery as follows (homeless vs non-homeless): cardiovascular/vascular (1 vs 2), respiratory (1 vs 1), gastrointestinal (1 vs 0), neurologic (1 vs 7), trauma (6 vs 2), and orthopedic (0 vs 2).

^4^Includes 2 patients admitted from the cardiac catheterization laboratory.

^5^Includes 1 patient admitted from the hospital’s dialysis unit.

^6^Patients were transferred from other hospitals with the following diagnoses: pancreatitis (n = 2), acute coronary syndrome requiring percutaneous cardiac intervention (n = 2), pericardial tamponade (n = 1), febrile neutropenia (n = 1), variceal bleed (n = 1), and drug overdose (n = 1).

^7^Ethanol, acetaminophen, and salicylate obtained from serum toxicology screens, and other substances from urine toxicology screens.

Table Abbreviations: APACHE II, Acute Physiology and Chronic Evaluation II score; EMS, emergency medical services; ICU, intensive care unit; MV, mechanical ventilation; n, number of patients.

The 63 homeless patients were successfully matched to 63 non-homeless control patients by year of hospital admission [all patients; with 50/63 (79%) matched pairs admitted within 60 days of each other], sex (all patients), age (exact match; except for 2 patients who were 1 year younger and 1 year older, respectively), and admitting ICU (all except one patient). We present baseline characteristics and data at ICU admission for both groups in Tables 1 and 2. We noted a higher proportion of homeless patients were brought to hospital by EMS (87% vs 65%, p = 0.006), and significantly more homeless patients were admitted directly through the emergency room (p = 0.02). Conversely, significantly more non-homeless patients were referred to our ICUs from other hospitals (p = 0.006). Documented comorbidities were similar except for a significantly lower prevalence of previously diagnosed hypertension in the homeless cohort (16% vs 40%, p = 0.005). While the median number of medications, among patients prescribed at least one medication, was similar between homeless and non-homeless patients, significantly fewer homeless patients were taking any prescription medications at baseline (48% vs 67%, p = 0.047) (S1 Table). History of smoking, alcohol use, and use of other illicit substances were all significantly higher in homeless patients. Compared to the non-homeless cohort, a higher proportion of homeless patients underwent toxicology drug screening on admission (75% vs 38%, p<0.001) but the proportion of screens that identified illicit substances was similar between groups (72% vs 71%, p = 1.00). A substantially higher proportion of homeless vs non-homeless patients’ toxicology screens detected the presence of alcohol (41% vs 10%, p<0.001). Illness acuity at ICU admission, as measured either by APACHE II score or need for mechanical ventilation, was non-significantly higher in the homeless cohort. Finally, significantly fewer homeless patients had a substitute decision-maker (SDM) identified at the time of admission compared to non-homeless patients (38% vs 83%; p<0.001).

### ICU clinical course (Table 3 and S2 and S3 Tables)

**Table 3:** ICU diagnostics and treatments.

Event or Treatment	Homeless (n = 63)	Not Homeless (n = 63)	p-value
Consults, mean ±SD	3.5 ±2.5	4.2 ±2.6	0.10
Enrollment in research study, n (%)	4 (6%)	4 (6%)	1.00
Diagnostic Tests, n (%)			
CT Scan	34 (54%)	30 (48%)	0.59
Echocardiogram	21 (33%)	20 (32%)	1.00
Ultrasound	12 (19%)	9 (14%)	0.63
MRI Scan	8 (13%)	7 (11%)	1.00
Invasive Procedures, n (%)			
Broncheoalveolar lavage	9 (14%)	7 (11%)	0.79
Angiography	4* (6%)	9 (14%)	0.24
Lumbar puncture	3 (5%)	2 (3%)	1.00
Esophagogastroduodenoscopy	1 (2%)	4 (6%)	0.36
Vasopressors/Inotropes	17 (27%)	12 (19%)	0.40
Vasopressor Days, median (IQR; range)	0 (0–1; 0–13)	0 (0–0; 0–6)	0.33
Vasopressor Days,** median (IQR; range)	3 (2–6; 1–13)	2 (1–2; 1–6)	0.06
Antibiotics	37 (59%)	37 (59%)	1.00
Antibiotic Days, median (IQR; range)	2 (0–6; 0–33)	2 (0–4; 0–17)	0.40
Antibiotic Days,** median (IQR; range)	5 (3–9; 1–33)	3 (2–6; 1–17)	0.06
Renal Replacement Therapy	5 (8%)	6 (10%)	0.99
Transfusion of Blood Products	14 (22%)	14 (22%)	1.00
Parenteral Nutrition	1 (2%)	1 (2%)	1.00
Other Medications			
Sedatives***	60 (95%)	57 (90%)	0.49
Thiamine	45 (71%)	13 (21%)	<0.0001
Nicotine Replacement Therapy	24 (38%)	9 (14%)	0.004
Corticosteroids	14 (22%)	17 (27%)	0.68

Notes.

*Includes 2 patients admitted from the cardiac catheterization laboratory.

** Only patients receiving these medications.

*** Sedatives refers to anxiolytics, anti-psychotics, and opioids.

Table Abbreviations: CT, computerized tomography; ICU, intensive care unit; IQR, interquartile range; MRI, magnetic resonance imaging; n, number of patients; SD, standard deviation.

We noted similar physiological variables, measured on ICU days 1, 3, and 7, between cohorts (S2 Table). However, Glasgow Coma Scale (GCS) scores were significantly lower in the homeless group on days 1 (8.8 vs 11.0, p = 0.006) and 3 (10.0 vs 11.8, p = 0.04), but not day 7 (7.6 vs 9.5, p = 0.10). While in ICU, homeless patients were assessed by similar numbers of consultation services, and underwent diagnostic tests (CT scan, echocardiography, MRI, and ultrasound) and invasive procedures (broncheoalveolar lavage, angiography, lumbar puncture, and esophagogastroduodenoscopy) with similar frequency (Table 3). Frequency of microbiologic testing and proportions of positive bacterial culture results were similar, with the exception of the proportion of patients with positive respiratory cultures, which was higher among homeless compared to non-homeless patients (38% vs 21%, p<0.05) (S3 Table). Although similar proportions of homeless vs non-homeless patients were treated with antibiotics and vasopressors/inotropes and for similar durations overall, treated patients had non-significantly longer median treatment durations. Similar proportions of homeless vs non-homeless patients received renal replacement therapy, parenteral nutrition, blood transfusions, and other medications (sedatives, corticosteroids), except for thiamine (71% vs 21%, p<0.0001) and nicotine replacement therapy (38% vs 14%, p = 0.004) which were both prescribed more frequently in homeless patients. Similarly low numbers of homeless and non-homeless patients (6% in each group) were enrolled in research studies in the ICU.

### ICU and hospital outcomes (Table 4)

**Table 4:** ICU and hospital outcomes.

Outcome	Homeless (n = 63)	Not Homeless (n = 63)	p-value
Ventilator days, mean ±SD	4.2 ±7.1	2.0 ±3.5	0.03
Ventilator days, median (IQR; range)	1.0 (0–5; 0–31)	0.5 (0–2; 0–14)	0.052
Re-intubation*, n/N (%)	3/44 (7%)	3/33 (9%)	1.00
ICU length of stay (days), mean ±SD	6.4 ± 7.6	4.1 ± 4.6	0.04
ICU length of stay (days), median (IQR; range)	3 (2–8; 1–32)	2 (1–5; 1–18)	0.07
Re-admission to ICU, n (%)	6 (10%)	4 (6%)	0.74
Hospital length of stay (days), mean ±SD	19.0 ±24.5	12.7 ± 12.5	0.07
Hospital length of stay (days), median (IQR; range)	11(5–24; 1–154)	8 (5–19; 1–61)	0.17
Advanced directive obtained during ICU stay, n (%)	7 (11%)	3 (5%)	0.32
Hospital mortality, n (%)	18 (29%)	5 (8%)	0.005
Process of Death, n (%)			1.00
Withdrawal of life sustaining therapy in ICU, n/N (%)	11/18 (61%)	3/5 (60%)	
Non-resuscitated cardiac arrest (ICU or Ward), n/N (%)	5/18 (28%)	1/5 (20%)	
No limitations in life sustaining therapies, n/N (%)	2/18 (11%)	1/5 (20%)	
End-of-life decision-making, n (%)			0.15
Family/SDM involvement, n/N (%)	9/18 (50%)	5/5 (100%)	
No family/SDM involvement**, n/N (%)	8/18 (44%)		
Known patient wishes, n/N (%)	1/18 (6%)		
Survivor disposition at discharge, n/N (%)			0.20
Home/shelter/jail	35/45 (78%)	38/58 (66%)	
Acute care hospital/LTC	10/45 (22%)	20/58 (34%)	

Notes.

* Calculated as a proportion of patients ever intubated.

** Decisions made jointly by the inter-professional team in consultation with the Public Guardian and Trustee when necessary (n = 5 on hospital day 9, 12, 31, 59, and 83).

Table Abbreviations: ICU, intensive care unit; IQR, interquartile range; LTC, long term care; n, number of patients; N, total number of patients; SD, standard deviation; SDM, substitute decision maker.

Homeless patients had non-significantly longer median duration of ventilation and lengths of ICU and hospital stay (p = 0.052, 0.07, and 0.17, respectively). Despite these findings, homeless patients had significantly higher hospital mortality (29% vs 8%, p = 0.005) with similar proportions of deaths occurring while receiving maximal therapy or due to withdrawal of life sustaining therapy. Whereas family members were involved in decisions for all (n = 5; 100%) deaths in non-homeless patients, family members could only be located for 9/18 (50%) homeless patient decedents (p = 0.15). The Office of the Public Guardian and Trustee in Ontario was involved in decisions regarding continuation or withdrawal of life sustaining therapy in over half (5/9) of homeless patients without an identifiable family member or SDM, and in these five patients, decisions to withdraw life-sustaining therapies were made on hospital day 9, 12, 31, 59, and 83, respectively. Detailed review of all deaths in both groups disclosed that withdrawal of life-sustaining therapy occurred in similar clinical circumstances—regardless of the familial involvement in decision-making. Among survivors, a lower proportion of homeless patients were discharged to other acute care or long-term care/rehabilitation hospitals but this difference did not reach statistical significance (22% vs 34%, p = 0.20).

We attempted to conduct multivariable regression analysis to determine if homelessness was an independent predictor of hospital mortality. With the small number of hospital mortality events (5 non-homeless and 18 homeless) and large number of potential explanatory variables, we found that we could create several different models, each containing different uncorrelated variables with high discriminatory power (area under the receiver operating curve >0.9). When homelessness was forced into the model, it appeared to be an independent predictor of hospital mortality. However, if homelessness was not forced into the model, it was not retained as a predictor.

## Discussion

Few studies have described the characteristics and clinical course of critically ill homeless patients. Our study represents the first attempt to compare the ICU clinical course and outcomes of homeless compared to non-homeless patients, matched for age, gender, and type of ICU, characteristics most commonly used by bedside clinicians. Compared to non-homeless patients, homeless patients had similar medical comorbidities but were much more likely to have a history of substance use. Differences in the proportion of patients with a previous diagnosis of hypertension and use of prescription medications at admission suggest under-diagnosis and under-treatment of chronic conditions in homeless persons, even within the Canadian system of universal health insurance. Severity of illness between homeless and non-homeless cohorts was similar at ICU admission and during the ICU stay. Critically ill homeless patients had similar rates of diagnostic testing, invasive procedures, treatments, and consults. Regarding physiological parameters, homeless patients had lower GCS scores at most time points in the ICU. Homeless patients were also more likely to have positive respiratory cultures for pathogenic bacteria. We observed non-significant trends toward longer duration of vasopressor/inotrope and antibiotic therapy in treated patients, as well as non-significant trends towards longer duration of ventilation and ICU and hospital stays. These latter factors are generally associated with increased morbidity and mortality, and may have contributed to the significantly higher hospital mortality in our homeless patient cohort.

Importantly, we found homeless patients in ICU did not undergo fewer investigations or receive fewer treatments compared to matched non-homeless patients. Comparable health care delivery in the ICU was achieved in the Canadian context of universal health coverage for citizens. After matching for age, sex, type of ICU, and noting that matched homeless patients and controls had similar baseline clinical characteristics with the exception of substance use, homeless patients experienced trends towards longer periods of mechanical ventilation, ICU and hospital stays. We hypothesize that these findings may relate to a greater tendency for substance withdrawal or increased agitation associated with chemical dependence. This hypothesis is, in part, supported by the significantly lower average GCS scores noted in homeless versus matched non-homeless study participants and higher use of nicotine replacement therapy to treat nicotine withdrawal; however, we did not systematically record agitation scores or withdrawal management. Alternatively, differences in lengths of stay may be associated with differences in unmeasured illness severity, or complications arising during the ICU stay such as respiratory infections, as suggested by higher rates of positive respiratory cultures and trends towards longer durations of antibiotic therapy and vasopressor/inotrope use in patients requiring these treatments. The higher rate of positive respiratory cultures may suggest increased predisposition to all types of pneumonia (community acquired, ventilator associated, and aspiration) or impaired mucociliary clearance from higher rates of smoking. The finding of high rates of positive respiratory cultures among homeless ICU patients is in keeping with previous studies affirming high rates of invasive pneumococcal disease in this population [14]. In our study 4 homeless vs 0 non-homeless patients had respiratory cultures positive for pneumococcus (p = 0.12).

Reasons for the higher hospital mortality among homeless compared to non-homeless ICU patients are not entirely clear. Underdiagnosis and unmeasured differences in the severity of comorbid conditions may have been contributing factors. In addition, higher rates of substance use leading to more decreased level of consciousness (either directly due to the substances, or secondary to sedative administration to treat substance withdrawal) may have contributed to trends towards greater duration of ventilation and ICU lengths of stay, and possibly a greater need for vasopressors either due to sedation-mediated hypotension or secondary infection from more prolonged ventilation which also may have also contributed to differences in outcomes. Due to small numbers of deaths, logistic regression did not definitively identify homelessness or other factors as independent predictors of mortality as multiple models with unrelated and different predictors could be generated with equal discriminatory power. Differences in mortality do not appear to be due to differential rates of withholding or withdrawing of life sustaining therapies.

A recently published propensity-matched cohort study conducted in France described the epidemiology and outcomes of critically ill homeless patients [12]. This study compared 421 homeless to 9,353 non-homeless patients admitted over a 12-year period. Baseline demographics were very similar to our homeless cohort, consisting of primarily (89%) male patients with median age of 49 years old, although a higher proportion of the patients in this study lived on the street (70%) rather than in shelters. Similar to our study, acuity of illness was comparable between homeless and non-homeless patients. However, in contrast to our study, these authors reported significantly longer ICU and hospital lengths of stay, but similar hospital mortality. The reasons for these differences in outcome are unclear, as the French study did not provide data on clinical course, treatments, or end-of-life decision making. The French study did include individuals living with family, or in hostels or hotels, whereas our study used what seems to be a more extreme definition of homelessness including only unsheltered or temporary emergency sheltered individuals, and this may partially explain the poorer outcomes observed in our study.

A small body of literature has addressed issues of poverty, socioeconomic status (SES), and ICU-related outcomes without specific reference to homelessness [11]. In a review of 38,917 ICU admissions at 2 academic hospitals in Boston, neighbourhood poverty rate was not associated with 30-day, 90-day, 365-day, or hospital mortality [15]. However, within this same cohort, patients living in neighborhoods with high poverty rates were significantly more likely to have positive blood cultures within 48 hours of admission to ICU [16]. Neighbourhood poverty rate was ascertained using residential addresses and thus individuals without a fixed address were systematically excluded from these studies. Consequently the generalizability of these findings to the homeless is uncertain. In a German study linking individual SES data to clinical outcomes in the ICU, low SES was associated with a greater risk of organ failure and prolonged ICU stay, and there was an inverse relationship between SES and severity of illness at ICU admission and ICU length of stay [17]. Similar to the Boston studies, it is unclear whether the study sample included homeless individuals.

### Strengths and limitations

Our study has several strengths. First, this study is novel in investigating the potential impact of homelessness on ICU outcomes in a variety of ICU types (medical/surgical, neurosurgical/trauma, cardiovascular, and cardiac) and is the first to report detailed data on ICU treatments and physiological parameters during the ICU stay and end-of-life decision making. Second, we matched patients and controls based on criteria thought to be of prognostic importance and were able to achieve near-perfect matches between cases and controls by age, sex, and type of ICU. Third, we verified each included case as homeless. Our study also has weaknesses. This is a single centre retrospective study subject to biases inherent in chart abstraction. We chose age and sex as our primary comparison criteria since these are most commonly used by clinicians when initially assessing a patient. We compared other comorbidities but did not formally match on these other criteria. The small number of included patients suggests the study had low statistical power to identify differences between groups. We did not formally correct p-values for multiple comparisons and testing; however, we prioritized differences for which p-values were highly significant similar to the philosophy of correction techniques. Finally, we used a binary classification of homelessness based on the index hospital admission, recognizing that homelessness is a dynamic state and that patients may be homeless for different periods of time. It is likely patients who are chronically homeless have different outcomes than patients who are only transiently homeless; such differences would not be identifiable in our study that was based on a single hospital admission where duration of homelessness was not recorded.

## Conclusions

In this cohort study matching homeless and non-homeless patients by admission ICU, age, and sex, information readily available to bedside clinicians, the groups exhibited similar comorbidities, with the exception of a lower rate of previously documented hypertension, increased substance use, and lower GCS scores among homeless patients. Despite similarities in illness severity at ICU admission, treatment intensity in our publically funded health care system, homeless patients had significantly higher hospital mortality. The reasons for the higher observed mortality are not entirely clear. These findings support the need for additional research to validate our findings in other health care settings and develop strategies to improve outcomes in this vulnerable population.

## Supporting information

S1 Table Pre-hospitalization medications. * In patients taking any medications representing 30 homeless and 42 non-homeless patients. ** Pain medication grouping includes acetaminophen and NSAIDs. Table Abbreviations: ACE, angiotensin converting enzyme; ARB, angiotensin II receptor blockers; HAART highly active anti-retroviral therapy; IQR, interquartile range; n, number of patients; NSAID non-steroidal anti-inflammatory drug; PPI proton pump inhibitors.
(DOCX)

S2 Table Physiological parameters during ICU stay. Denominators range from 5–63 due to missing values and due to discharge/death from ICU. * Range of lowest Glasgow Coma Scale scores was 3–15 for both homeless and not homeless groups on each ICU Day 1, 3, and 7. Table Abbreviations: ICU, intensive care unit; IQR, interquartile range; n, number of patients; N, total number of patients; PF, ratio of arterial oxygen partial pressure to fractional inspired oxygen; SD, standard deviation.
(DOCX)

S3 Table Microbiology testing and results 48 hours before and after ICU admission. * Two or more organisms growing in a single culture specimen (from any site); ** Positive cultures from two or more different sites; ***Any patient meeting this criterion also meets criterion for “Multiple Positive cultures”. Positive respiratory cultures were defined based on final microbiologic results available in the patient charts as per the standards of our microbiology laboratory. Sputum gram stains were also performed but gram stain results were not used to classify results as positive or negative. Culture and sensitivity results were analyzed as per standard clinical microbiology practices applied in our laboratory. BALs were analyzed quantitatively and considered positive when there were ≥10^4^ CFU/mL. None of our patients had protected brush samples. ETT and bronchial wash cultures were analyzed semi-quantitatively based on the extent of growth on culture media and classified as "positive" for this study if pathogens were reported with full identification and susceptibility results. Table Abbreviations: BAL, bronchioalveolar lavage; CFU, colony forming units; ETT, endotracheal tube; ICU, intensive care unit; MRSA, *methicillin resistant staphylococcus aureus*; MSSA, *methicillin sensitive staphylococcus aureus*; n, number of patients.
(DOCX)
