# Supplementary material for: Characteristics, clinical course, and outcomes of homeless and non-homeless patients admitted to ICU: A retrospective cohort study
Source: PLoS One. 2017 Jun 12;12(6):e0179207. doi: 10.1371/journal.pone.0179207 (PMC5467852; doi:10.1371/journal.pone.0179207)
Supplement: S1 Table — * In patients taking any medications representing 30 homeless and 42 non-homeless patients. ** Pain medication grouping includes acetaminophen and NSAIDs. Table Abbreviations: ACE, angiotensin converting enzyme; ARB, angiotensin II receptor blockers; HAART highly active anti-retroviral therapy; IQR, interquartile range; n, number of patients; NSAID non-steroidal anti-inflammatory drug; PPI proton pump inhibitors. (DOCX) [file pone.0179207.s001.docx]

**S1 Table. Pre-hospitalization Medications**

| **Characteristic** | **Homeless (n=63)** | **Not Homeless (n=63)** | **p-value** |
| --- | --- | --- | --- |
| Number of medications, median (IQR; range)  Number of medications*, median (IQR; range) | 0 (0-4.5; 0-18)  5 (2-10; 1-18) | 2 (0-6; 0-14)  5 (2-8; 1-14) | 0.18  0.83 |
| **Type of medication, n (%)** |  |  |  |
| *Cardiovascular* |  |  |  |
| ACE inhibitors/ARB | 11 (17%) | 16 (25%) | 0.39 |
| Cholesterol lowering medications | 7 (11%) | 12 (19%) | 0.31 |
| Diuretics | 7 (11%) | 5 (8%) | 0.76 |
| Beta-blockers | 6 (10%) | 13 (21%) | 0.13 |
| Anti-platelet agents | 6 (10%) | 11 (17%) | 0.30 |
| Calcium channel blockers | 5 (8%) | 9 (14%) | 0.40 |
| Anti-diabetic (oral or insulin) | 4 (6%) | 5 (8%) | 1.00 |
| Other cardiovascular | 2 (3%) | 6 (10%) | 0.27 |
| Oral anticoagulants | 1 (2%) | 4 (6%) | 0.36 |
| *Neurological* |  |  |  |
| Anti-psychotics | 16 (25%) | 9 (14%) | 0.18 |
| Pain medications** | 8 (13%) | 5 (8%) | 0.56 |
| Anti-epileptics | 9 (14%) | 2 (3%) | 0.054 |
| Opioids | 7 (11%) | 12 (19%) | 0.32 |
| Benzodiazepines | 4 (6%) | 7 (11%) | 0.53 |
| *Other* |  |  |  |
| PPI | 11 (17%) | 5 (8%) | 0.18 |
| Antibiotics | 9 (14%) | 7 (11%) | 0.79 |
| Minerals/vitamins | 8 (13%) | 7 (11%) | 1.00 |
| Other gastrointestinal | 6 (10%) | 6 (10%) | 1.00 |
| Inhalers | 3 (5%) | 4 (6%) | 1.00 |
| Steroids | 2 (3%) | 2 (3%) | 1.00 |
| Thyroid | 0 | 5 (8%) | 0.058 |
| HAART | 0 | 4 (6%) | 0.12 |

*Notes.* * In patients taking any medications representing 30 homeless and 42 non-homeless patients*.* ** Pain medication grouping includes acetaminophen and NSAIDs.

Table Abbreviations: ACE, angiotensin converting enzyme; ARB, angiotensin II receptor blockers; HAART highly active anti-retroviral therapy; IQR, interquartile range; n, number of patients; NSAID non-steroidal anti-inflammatory drug; PPI proton pump inhibitors.
