# Supplementary material for: Characteristics, clinical course, and outcomes of homeless and non-homeless patients admitted to ICU: A retrospective cohort study
Source: PLoS One. 2017 Jun 12;12(6):e0179207. doi: 10.1371/journal.pone.0179207 (PMC5467852; doi:10.1371/journal.pone.0179207)
Supplement: S2 Table — Denominators range from 5–63 due to missing values and due to discharge/death from ICU. * Range of lowest Glasgow Coma Scale scores was 3–15 for both homeless and not homeless groups on each ICU Day 1, 3, and 7. Table Abbreviations: ICU, intensive care unit; IQR, interquartile range; n, number of patients; N, total number of patients; PF, ratio of arterial oxygen partial pressure to fractional inspired oxygen; SD, standard deviation. (DOCX) [file pone.0179207.s002.docx]

**S2 Table. Physiological Parameters During ICU Stay**

| **Clinical Variable** | **Homeless**  **Mean ±SD; Median (IQR) or n/N (%)** | **Not Homeless**  **Mean ±SD; Median (IQR) or n/N (%)** | **p-value** |
| --- | --- | --- | --- |
| **ICU Day 1**  Heart rate (Maximum)  Mean arterial pressure (Minimum)  PF ratio (lowest)  Number on vasopressors, n/N (%)  Receipt of blood product, n/N (%)  Glasgow Coma Scale (Lowest)*  Platelets (Lowest)  Total bilirubin (Highest)  Creatinine (Highest) | 106 ±30; 108 (86-125)  86 ±26; 82 (62-109)  256 ±129; 272 (150-350)  9/63 (14%)  6/63 (10%)  8.8 ±4.4; 8.5 (5-14)  193 ±133; 173 (126-234)  25 ±30; 14 (12-29)  111 ±143; 72 (59-102) | 92 ±29; 99 (65-115)  80 ±24; 70 (63-100)  243 ±113; 210 (158-349)  9/63 (14%)  6/63 (10%)  11.0 ±4.4; 13 (7-15)  164 ±72; 157 (128-208)  26 ±27; 17 (13-30)  146 ±182; 85 (71-122) | 0.02  0.21  0.79  1.00  1.00  0.006  0.27  0.39  0.06 |
| **ICU Day 3**  Heart rate (Maximum)  Mean arterial pressure (Minimum)  PF ratio (lowest)  Number on vasopressors, n/N (%)  Receipt of blood product, n/N (%)  Glasgow Coma Scale (Lowest)*  Platelets (Lowest)  Total bilirubin (Highest)  Creatinine (Highest) | 103 ±32; 108 (73-125)  87 ±26; 83 (65-107)  242 ±106; 220 (160-296)  9/52 (17%)  1/52 (2%)  10.0 ±4.5; 10 (7-15)  158 ±80; 151 (107-218)  43 ±55; 23 (15-29)  122 ±158; 70 (49-126) | 104 ±33; 108 (88-121)  87 ±29; 76 (65-113)  247 ±95; 239 (165-310)  4/46 (9%)  4/46 (9%)  11.8 ±3.8; 14 (9-15)  138 ±77; 106 (91-166)  34 ±36; 18 (13-33)  150 ±154; 81 (63-159) | 0.96  0.83  0.66  0.25  0.18  0.04  0.21  0.61  0.11 |
| **ICU Day 7**  Heart rate (Maximum)  Mean arterial pressure (Minimum)  PF ratio (lowest)  Number on vasopressors, n/N (%)  Receipt of blood product, n/N (%)  Glasgow Coma Scale (Lowest)*  Platelets (Lowest)  Total bilirubin (Highest)  Creatinine (Highest) | 113 ±35; 120 (84-129)  81 ±23; 72 (64-102)  225 ±91; 210 (159-269)  3/23 (13%)  4/23 (17%)  7.6 ±3.8; 8 (4-9)  233 ±122; 237 (161-319)  83 ±96; 75 (12-95)  130 ±196; 56 (40-105) | 94 ±29; 108 (67-117)  104 ±27; 107 (78-126)  240 ±109; 263 (164-326)  0/14  1/14 (7%)  9.5 ±3.7; 10 (9-11)  213 ±70; 197 (167-273)  16 ±9; 12 (10-23)  84 ±71; 78 (41-83) | 0.09  0.01  0.67  0.27  0.63  0.10  0.42  0.18  0.87 |

*Notes*. Denominators range from 5 – 63 due to missing values and due to discharge/death from ICU. * Range of lowest Glasgow Coma Scale scores was 3-15 for both homeless and not homeless groups on each ICU Day 1, 3, and 7.

Table Abbreviations: ICU, intensive care unit; IQR, interquartile range; n, number of patients; N, total number of patients; PF, ratio of arterial oxygen partial pressure to fractional inspired oxygen; SD, standard deviation.
