# Supplementary material for: Characteristics, clinical course, and outcomes of homeless and non-homeless patients admitted to ICU: A retrospective cohort study
Source: PLoS One. 2017 Jun 12;12(6):e0179207. doi: 10.1371/journal.pone.0179207 (PMC5467852; doi:10.1371/journal.pone.0179207)
Supplement: S3 Table — * Two or more organisms growing in a single culture specimen (from any site); ** Positive cultures from two or more different sites; ***Any patient meeting this criterion also meets criterion for “Multiple Positive cultures”. Positive respiratory cultures were defined based on final microbiologic results available in the patient charts as per the standards of our microbiology laboratory. Sputum gram stains were also performed but gram stain results were not used to classify results as positive or negative. Culture and sensitivity results were analyzed as per standard clinical microbiology practices applied in our laboratory. BALs were analyzed quantitatively and considered positive when there were ≥104 CFU/mL. None of our patients had protected brush samples. ETT and bronchial wash cultures were analyzed semi-quantitatively based on the extent of growth on culture media and classified as "positive" for this study if pathogens were reported with full identification and susceptibility results. Table Abbreviations: BAL, bronchioalveolar lavage; CFU, colony forming units; ETT, endotracheal tube; ICU, intensive care unit; MRSA, methicillin resistant staphylococcus aureus; MSSA, methicillin sensitive staphylococcus aureus; n, number of patients. (DOCX) [file pone.0179207.s003.docx]

**S3 Table. Microbiology Testing and Results 48 Hours Before and After ICU Admission**

| **Variable** | **Homeless**  **(n=63)** | **Not homeless**  **(n=63)** | **p- value** |
| --- | --- | --- | --- |
| Microbiology testing, n (%) | 33 (52%) | 26 (41%) | 0.28 |
| Any positive culture, n (%) | 32 (51%) | 23 (37%) | 0.15 |
| Any polymicrobial* culture, n (%) | 11 (17%) | 5 (8%) | 0.18 |
| Multiple** positive cultures, n (%) | 13 (21%) | 8 (13%) | 0.34 |
| Positive blood culture AND at least one positive culture from another site***, n (%) | 6 (10%) | 4 (6%) | 0.74 |
| Positive blood culture, n (%) | 9 (14%) | 6 (10%) | 0.58 |
| Positive respiratory culture, n (%) | 24 (38%) | 13 (21%) | 0.0497 |
| *Gram Positive Bacteria* |  |  |  |
| MRSA | 5 | - |  |
| MSSA | 4 | 2 |  |
| Streptococcus pneumonia | 4 | - |  |
|  |  |  |  |
| *Gram Negative Bacteria* |  |  |  |
| Escherichia coli | 6 | - |  |
| Haemophilus influenza | 3 | 1 |  |
| Moraxella catarrhalis | 2 | 2 |  |
| Klebsiella pneumonia | 1 | 2 |  |
| Enterobacter cloacae | - | 2 |  |
| Citrobacter koseri | 1 | - |  |
| Pseudomonas aeruginosa | 1 | 1 |  |
| Serratia marcesans | 1 | - |  |
| Eikenella corrodens | - | 1 |  |
|  |  |  |  |
| *Mycobacterium Species* | 2 | 1 |  |
|  |  |  |  |
| *Fungal* |  |  |  |
| Candida albicans | 4 | 1 |  |
| Rhizopus microspores | 1 | - |  |
| Pneumocystis jirovecii | - | 2 |  |
| Aspergillus | - | 1 |  |
|  |  |  |  |
| *Viruses* |  |  |  |
| Adenovirus | 2 | - |  |
| Herpes Simplex Virus | 1 | - |  |
|  |  |  |  |
| Positive gastrointestinal culture, n (%) | 3 (5%) | 2 (3%) | 1.00 |
| Positive genitourinary culture, n (%) | 6 (10%) | 7 (11%) | 1.00 |
| Positive swab culture, n (%) | 11 (17%) | 6 (10%) | 0.30 |

*Notes.* * Two or more organisms growing in a single culture specimen (from any site); ** Positive cultures from two or more different sites; ***Any patient meeting this criterion also meets criterion for “Multiple Positive cultures”.

Positive respiratory cultures were defined based on final microbiologic results available in the patient charts as per the standards of our microbiology laboratory. Sputum gram stains were also performed but gram stain results were not used to classify results as positive or negative. Culture and sensitivity results were analyzed as per standard clinical microbiology practices applied in our laboratory. BALs were analyzed quantitatively and considered positive when there were ≥10^4^ CFU/mL. None of our patients had protected brush samples.  ETT and bronchial wash cultures were analyzed semi-quantitatively based on the extent of growth on culture media and classified as "positive" for this study if pathogens were reported with full identification and susceptibility results.

Table Abbreviations: BAL, bronchioalveolar lavage; CFU, colony forming units; ETT, endotracheal tube; ICU, intensive care unit; MRSA*, methicillin resistant staphylococcus aureus;* MSSA, *methicillin sensitive staphylococcus aureus*; n, number of patients*.*
